# Cortactin: A Major Cellular Target of the Gastric Carcinogen *Helicobacter pylori*

**DOI:** 10.3390/cancers12010159

**Published:** 2020-01-09

**Authors:** Irshad Sharafutdinov, Steffen Backert, Nicole Tegtmeyer

**Affiliations:** Division of Microbiology, Department of Biology, Friedrich Alexander University Erlangen-Nuremberg, Staudtstr. 5, D-91058 Erlangen, Germany; irshad.sharafutdinov@fau.de (I.S.); steffen.backert@fau.de (S.B.)

**Keywords:** Abl, Src, CagA, *cag*PAI, FAK, *Helicobacter pylori*, signaling, type IV secretion, T4SS, tyrosine kinases, SHP2

## Abstract

Cortactin is an actin binding protein and actin nucleation promoting factor regulating cytoskeletal rearrangements in nearly all eukaryotic cell types. From this perspective, cortactin poses an attractive target for pathogens to manipulate a given host cell to their own benefit. One of the pathogens following this strategy is *Helicobacter pylori*, which can cause a variety of gastric diseases and has been shown to be the major risk factor for the onset of gastric cancer. During infection of gastric epithelial cells, *H. pylori* hijacks the cellular kinase signaling pathways, leading to the disruption of key cell functions. Specifically, by overruling the phosphorylation status of cortactin, *H. pylori* alternates the activity of molecular interaction partners of this important protein, thereby manipulating the performance of actin-cytoskeletal rearrangements and cell movement. In addition, *H. pylori* utilizes a unique mechanism to activate focal adhesion kinase, which subsequently prevents host epithelial cells from extensive lifting from the extracellular matrix in order to achieve chronic infection in the human stomach.

## 1. Introduction

The human pathogen *Helicobacter pylori* currently colonizes the stomach of over half of the world’s population. Although the bacteria can be present in the gastric environment asymptomatically for years or even decades, their presence contributes to the development of gastric disorders such as gastritis, peptic ulcers, and stomach cancer in a subset of persons [1,2,3,4]. This is the result of the bacterial virulence machinery hijacking the host’s defense capacity, as the bacteria can invade the protective epithelial cell layer of the stomach [5]. Approximately 10–20% of infected individuals eventually develop ulcer disease, while 1–2% will develop distal gastric cancer and <1% of infections result in mucosa-associated lymphoid tissue (MALT) lymphoma [4,6]. *H. pylori* can trigger signal activation of an otherwise constitutively expressed epidermal growth factor receptor (EGFR), which then can initiate neoplastic transformation by acceleration of cell proliferation and cell migration [7,8,9]. In addition, *H. pylori* infection induces cancer-related DNA damage and proteasomal degradation of p53, the guardian of genome stability [10,11,12]. When the pathogen is eradicated by antibiotic treatment, MALT lymphoma regresses in over 75% of cases, suggesting that continuous presence of the bacteria is required to maintain malignancy potential [13,14]. In addition, eradication of the pathogen significantly reduces the chance of recurring gastritis and peptic ulceration [15,16]. According to 2018 estimates by the World Health Organization (WHO) and Global Burden of Cancer Study (GLOBOCAN), gastric cancer is the third leading cause of annual deaths due to cancer worldwide [17]. Gastric cancer typically has a poor prognosis as metastases have often developed by the time of discovery. *H. pylori* typically causes non-cardia gastric cancer, for which peritoneal metastases are more common, in contrast to non-cardia cancer types [18]. A Swedish nationwide investigation has shown that most metastases from gastric cancer are detected in the liver (found in 48% of metastatic cancer patients), followed by the peritoneum (32%), lungs (15%), and bone (12%) [19].

During infection, *H. pylori* targets, amongst others, the cellular protein cortactin that is crucial for correct regulation of cytoskeletal rearrangements in healthy cells. De-regulation of cortactin activity in the cell plays a crucial role in the development of various forms of cancer as well as non-malignant disorders such as inflammatory bowel disease [20]. It is becoming apparent that *H. pylori* can contribute to the development of various gastric diseases through the modulation of cortactin’s binding partners and their activity. Here, we review these cortactin activities and the signaling pathways that contribute to the pathogenesis of *H. pylori.* Before dealing with the pathogen-induced malfunctioning of cortactin, its natural activity is first summarized.

## 2. Cortactin Activity Depends on Its Phosphorylation States and Is Involved in Tumor Development

**Figure 1:**
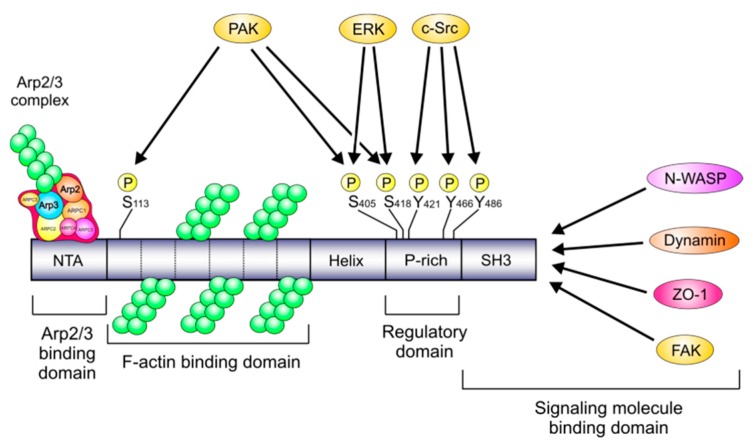
Simplified model of the cortactin protein due to cell infection with *Helicobacter pylori*: domain structure, phosphorylation sites, and interaction partners. The NTA and F-actin binding domains of cortactin provide stabilization of the Arp2/3-mediated F-actin branches. The prolin-rich domain harbors phospho-sites for the tyrosine and serine/threonine kinases ERK1/2, PAK, and c-Src; however, the target S-113 for PAK kinase is located in the “cortactin repeat” domain. The carboxy-terminal SH3 domain of cortactin has been shown to interact with more than 10 proteins [23,24,26,27,31,32,33,45,46,47,48,49,50], four of which, namely N-WASP, dynamin, ZO-1 and FAK have been shown to be affected during *H. pylori* infection [24,51,52,53]. However, the interaction of ZO-1 and dynamin with cortactin during *H. pylori* infection remains not clear.

Cortactin is a multidomain protein consisting of an N-terminal acidic domain (NTA) followed by a filamentous actin (F-actin) binding region, a proline-rich domain, and a C-terminal Src homology 3 (SH3) domain [21,22] (Figure 1). When analyzed by denaturing polyacrylamide gel electrophoresis (SDS-PAGE), cortactin produces two bands at approximately 80 and 85 kDa which have been named p80 and p85, respectively and they represent two populations of the protein [23,24]. Under normal conditions, the NTA domain of cortactin interacts, by means of a DDW motif, with the Arp2/3 protein complex which subsequently activates actin polymerization. Its F-actin binding region contains 6.5 copies of so called “cortactin repeats” that directly bind to F-actin. Both the NTA and the F-actin binding domains are required for correct regulation of branched actin assembly [22]. Of importance is the proline-rich domain in cortactin that contains multiple phosphorylation sites, in particular the tyrosine residues Y-421, Y-470, and Y-486 in human cortactin (which correspond to Y-421, Y-466, and Y-482 in mouse cortactin) and the serine residues S-405 and S-418 [24,25]. Finally, the SH3 domain located toward the C-terminus interacts with proline-rich regions of other proteins [21]. The latter include Wiskott-Aldrich syndrome protein (N-WASP), WASP-Interacting Protein (WIP), myosin light chain kinase (MLCK), dynamin-1 and dynamin-2, and many others [20,26,27,28,29,30]. These are summarized in Table 1.

Regulation of cortactin activity is mediated by the phosphorylation of its tyrosine or serine residues by tyrosine and serine/threonine kinases, respectively (Figure 1). These include sarcoma kinase (Src), extracellular signal regulated kinases 1 and 2 (ERK1/2), and p21-activated kinase (PAK1) [35,42,54]. Non-phosphorylated cortactin is a globular protein whose structure is maintained by interactions between the SH3 domain and either intermolecular helical domains or the F-actin binding domain [55]. Upon phosphorylation, a conformational change is induced that acts as a molecular switch, as the SH3 domain is available for interaction with other factors [55]. As is proposed in the so-called “S-Y switch” model, Src phosphorylation of residues Y-421, Y-466, and Y-482 inhibits the ability of ERK phosphorylated cortactin (S-405 and S-418) to bind and activate the actin-nucleation promoting factor N-WASP [25,56]. However, the model is still incomplete. For instance, it remains controversial whether the ability of cortactin to crosslink F-actin is affected by its alternative phosphorylation states. Moreover, it has been shown that cortactin can also be phosphorylated at Y-421 and Y-466 through recruitment of the adaptor protein Nck1, and this provides an alternative pathway to activate N-WASP [40]. Accordingly, an alternative model was proposed that suggests both serine and tyrosine phosphorylation events may lead to the activation of N-WASP. In that model, S-405/S-418 phosphorylation is required for dominant lamellipodia dynamics, whereas tyrosine phosphorylation has no effect [57]. Irrespective of these molecular models, there is no doubt that phosphorylation of cortactin by certain tyrosine kinases results in phenotypic changes that include protrusion, invasion, and migration of the cells [43,58]. For instance, using *Aplysia* (seaslugs) as a model organism, it has been shown that Src-mediated tyrosine phosphorylation of cortactin at Y-499 regulates filopodia formation in the bag cell neurons of these organisms [44]. In human pancreatic ductal adenocarcinoma (PDAC) cells, Y-421 phosphorylation leads to an increased capacity to migrate, while cell migration can be reduced by the Src family kinase inhibitor dasatinib [39].

Protein acetylation is another major post-translational modification, in which the acetyl group from acetyl coenzyme A is transferred to a specific site on a given target protein [59]. Cortactin can be phosphorylated and also acetylated at its lysine residues, and acetylation reduces its capacity to bind F-actin [60,61]. By using double knockout cells that were experimentally co-transfected with both Src and cortactin, it was demonstrated that phosphorylation of cortactin’s tyrosine residues promotes deacetylation and inhibits cell spreading [62]. In conclusion, cortactin is a pivoting factor where signal transduction and cytoskeletal organization converge, as it regulates various actin-based cellular processes including cell invasion, cell migration, and tumor cell metastasis [43]. Multiple observations have evidenced that cortactin expression is increased in various types of human cancers, including gastric cancer, colorectal carcinoma, pancreatic cancer, head and neck squamous carcinomas (HNSCC), hepatocellular cancer, breast cancer and ovarian cancers [39,63,64,65,66,67,68]. Cortactin expression can be enhanced as a result of amplification of chromosomal band 11q13 [69] that harbors the cortactin-coding gene *cttn*. Notably, *cttn* amplification was associated with recurrence and reduced life expectancy in HNSCC patients [70]. Cortactin promotes cell motility and tumor metastasis via activation and stabilization of the Arp2/3 complex, which leads to the development of protrusive structures (invadopodia and lamellipodia) and subsequent degradation of the extracellular matrix (ECM) [21]. Here, we review the cortactin activities and signaling pathways contributing significantly to the pathogenesis of *H. pylori*.

## 3. *H. pylori* Virulence Factors

Before zooming in on the role of *H. pylori* in cortactin-dependent cell signaling, its virulence factors and their role in pathogenicity during infection are briefly summarized first. Infection of the gastric epithelial cell layer by *H. pylori* is the coordinated result of dozens of bacterial proteins that in concert provide the basis of bacterial cell adhesion, invasion, and interaction with the host immune system [1,2,3,4,71]. These include secreted urease that locally neutralizes the gastric acidic pH and the blood group antigen binding protein A (BabA) that allows the bacteria to adapt to shifts in acidity [72]. A number of bacterial outer membrane proteins function as adhesins, including BabA, sialic acid binding protein A (SabA), *Helicobacter* outer membrane protein Q (HopQ), and outer-inflammatory protein A (OipA). These proteins allow the bacteria to adhere onto the surface of epithelial cells. Three major virulence factors act intracellularly by specifically disrupting cell signaling and cell integrity: cytotoxin-associated gene A (CagA), vacuolating cytotoxin A (VacA), and serine protease high temperature requirement A (HtrA) [73,74,75,76]. Virulence depends on presence of the *cag* pathogenicity island (*cag*PAI), a genome island that encodes a type IV secretion system (T4SS). This secretion system is required to translocate CagA into the human gastric epithelial cells [71,77]. Once inside the cell, CagA initiates various signaling cascades by disrupting cell signaling networks that can lead to cytoskeletal rearrangements, altered cell polarity, and localized disruption of the epithelial barrier [11,78]. The VacA toxin (a pore-forming toxin) affects both epithelial cells and immune cells, including T cells, B cells, and macrophages [79]. This virulence factor dampens apoptosis and autophagy, while it promotes immune tolerance and as such enables persistent infection [73]. In addition, virulence factor HtrA has serine protease activity that attacks the cellular junctions of gastric epithelial cells [80,81]. After secretion into the epithelial layer, its substrates include occludin and claudin-8 of tight junctions, and E-cadherin of adherence junctions [5,82]. In concert these events allow *H. pylori* to cross the epithelial barrier and invade the intercellular space between epithelial cells to reach basal surfaces and the lamina propria [83].

## 4. *H. pylori* Affects Host Kinases that Control Cortactin

**Figure 2:**
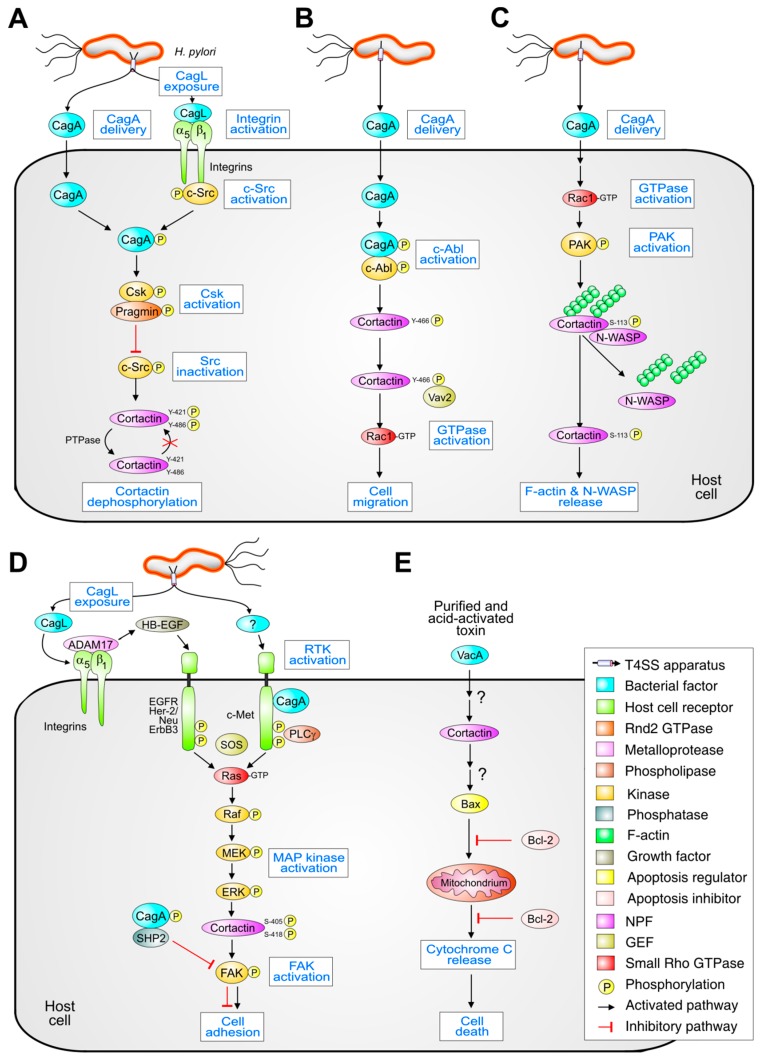
Schematic overview of molecular *H. pylori* signaling pathways that involve cortactin during infection of gastric epithelial cells. (**A**) The *H. pylori* T4SS pilus protein CagL activates c-Src tyrosine kinase through α_5_β_1_-integrin interaction [90]. Activated c-Src phosphorylates injected CagA, which in turn leads to the activation of Csk, a c-Src inhibitor [24,85]. Csk in complex with pragmin, a protein which potentiates kinase activity of Csk, inactivate c-Src that further results in tyrosine dephosphorylation of cortactin at Y-421 and Y-486 [91]. (**B**) The *H. pylori* protein CagA after injection into a host cell by the T4SS machinery activates tyrosine kinase c-Abl, which phosphorylates cortactin at Y-466 [92,93]. The downstream signaling leads to the recruitment of the Rho-family guanine nucleotide exchange factor Vav2 by phosphorylated cortactin, finally resulting in Rac1-mediated cell movement [94]. (**C**) Alternatively, CagA-activated Rac1 can lead to the activation of the serine-threonine kinase PAK, which phosphorylates cortactin at S-113 [24]. This leads to the dissociation of cortactin from the complex with F-actin and N-WASP. (**D**) During *H. pylori* infection, CagL dissociates ADAM17 from the α_5_β_1_-integrin [95]. Released ADAM17 increases the production of heparin-binding epidermal growth factor (HB-EGF), activating EGF receptor. Alternatively, *H. pylori* can activate hepatocyte growth factor receptor (HGFR) c-Met by a yet unknown factor, enhanced by interaction of CagA and signal transducing protein PLCγ [96]. The downstream signaling from either EGF or HGF receptors activates the Ras/ERK pathway, which leads to serine phosphorylation of cortactin at S-405 and S-418 [24]. Serine phosphorylated cortactin activates FAK, that results in increased cell adhesion [24]. In contrast, CagA-activated SHP2 phosphatase can downregulate activity of FAK due to SHP2-mediated dephosphorylation of FAK [97]. (**E**) Finally, high protein levels of cortactin were described to contribute to the apoptotic function of VacA in gastric epithelial cells in a yet unknown fashion [98]. Downstream signaling upregulates the pro-apoptotic protein Bax and downregulates the anti-apoptotic protein Bcl-2, which results in apoptosis of target cells.

As summarized above, the function of cortactin mainly depends on phosphorylation by tyrosine or serine/threonine kinases, while unphosphorylated cortactin is normally sequestered in the cytosol [84]. Upon infection, *H. pylori* affects the cortactin binding partners, which are mainly recruited to control cell attachment, movement and apoptosis (Figure 2). *H. pylori* injects CagA into the cellular cytosol by means of its T4SS, after which CagA hijacks cellular tyrosine kinases that control the behavior of many proteins, including cortactin. As has been shown by in vitro infection of AGS gastric epithelial cells, both Src and Abl kinases are activated by *H. pylori* in a specific and time-dependent fashion, which then phosphorylate CagA [58]. At an early stage of infection (during the first 1–2 h) activation of c-Src results from activity of the T4SS pilus-associated protein CagL, which binds to integrin-β1 triggering the phosphorylation of c-Src at Y-418 (Figure 2A). This phosphorylation increases the kinase activity of c-Src towards cortactin, as well as towards the *H. pylori* virulence factor CagA. This protein contains multiple phosphorylation domains (so-called EPIYA-repeats) that serve as substrates for c-Src [85]. In its phosporylated form, CagA directly binds c-Src and inactivates the enzyme via a negative feedback-loop that involves dephosphorylation of Y-418 and phosphorylation of the negative regulatory site Y-527. Phosphorylation of Y-527 is achieved by CagA-mediated binding and activation of carboxy-terminal Src kinase (Csk) through interaction of its PY-SH2 domain with phosphorylated CagA (Figure 2A). The enzyme responsible for dephosphorylation of Y-418 in Src is not yet known, but involvement of SHP2 can be excluded [86]. At later time points of infection (between 2 and 6 h), *H. pylori* continuously activates Abl kinases, which contrasts with the temporary activation of c-Src (Figure 2B, top). This activation of Abl is the result of its phosphorylation at position Y-412 and the enzyme then continues to phosphorylate CagA during the course of infection, while it can also phosphorylate the adapter protein CrkII at Y-221.

According to the molecular switch model described above, ERK needs to simultaneously phosphorylate both serine residues to fully activate cortactin. Interestingly, it was reported that during infection *H. pylori* could activate the MEK-ERK pathway in a time- and strain-dependent manner [87,88,89]. The activation of serine/threonine kinases PAK1 and ERK1/2, combined with the inactivation of c-Src, strictly correlates with the elongation phenotype that *H. pylori* induces between 4 and 8 h of infection [24]. Staining with activation-specific phospho-antibodies has revealed that both PAK1 (phosphorylated in T-423) and ERK1/2 (phosphorylated at both T-202 and Y-204) activity is increased continuously during infection, whereas activity of c-Src (phosphorylated at Y-416) is transient: while being activated for 0.5–2 h it is subsequently inactivated [24]. The *H. pylori*–dependent Src inactivation combined with the activation of ERK and PAK1 has enormous consequences for the host cell, as discussed below.

## 5. Role of Tyrosine-Phosphorylated Cortactin Generated by *H. pylori*

The effects of tyrosine phosphorylation on cortactin structure and function are still not completely understood, especially with regard to the activation of the actin-nucleation promoting factor N-WASP [56,57]. Cortactin has first been shown to be tyrosine phosphorylated during *H. pylori* infection in polarized Caco-2 cells, but the exact phosphosites remained unclear [93]. The next studies have revealed, that phosphorylation at Y-421, Y-466, and Y-482 is associated with a decrease in cortactin binding to F-actin [42], that was also observed in non-infected AGS cells, where cortactin was phosphorylated at all three sites [24,94]. Previous studies led to the assumption that all three tyrosine residues are dephosphorylated during *H. pylori* infection [24,85]. However, recently, we have shown that Y-421 and Y-482, but not Y-466, are dephosphorylated in infected AGS cells [94]. Recent work has further demonstrated that in breast cancer cells protein-tyrosine phosphatase 1B (PTP1B) regulates the phosphorylation status at Y-421 of cortactin during invadopodium precursor assembly [99]. In these cancer cells, invadopodium precursors recruit Mena^INV^ (an isoform of mammalian Ena protein), which promotes phosphorylation of cortactin at Y-421. In addition, phosphatase PTP1B, which can dephosphorylate this site, is suppressed so that cortactin remains phosphorylated at Y-421. This leads to recruitment of an Nck1/N-WASP/Arp2/3 complex that is essential for invadopodium maturation into a matrix-degrading structure [99]. In support of these observations, it was shown that in colon cancer cells, curcumin-mediated activation of PTP1B led to cortactin dephosphorylation at Y-421, resulting in reduced cell motility in colon cancer [100]. Thus, phosphorylation of cortactin at Y-421 seems to be necessary for cell migration in colon tumorigenesis.

This is where a role of *H. pylori* in oncogenesis can be proposed. We found that phosphorylation of cortactin at Y-466 is stabilized by *H. pylori* and this form of cortactin localizes in the cytosol of infected AGS cells (Figure 2B, bottom). Cortactin phosphorylated at Y-466 can then induce the interaction with the SH2 domain of the Rho-family guanine nucleotide exchange factor Vav2 [94]. Vav2 in turn becomes activated and induces small GTPase Rac1-mediated actin polymerization [94]. This signaling resulted in AGS cell movement and scattering, which is a hallmark of *H. pylori*–infected epithelial cells and was proposed to play a role in gastric carcinogenesis and metastasis [4,11]. Interestingly, cortactin phosphorylated at Y-421 and Y-466, but not at Y-482, was previously shown to recruit Vav2 and Rac3, promoting invadopodial maturation in invasive breast cancer cells [41]. The difference in the preference for binding of Rac1 versus Rac3 might be explained by cell type–specific differences in modification of cortactin and/or Vav2, which should be investigated in future studies.

## 6. Function of Serine-Phosphorylated Cortactin by *H. pylori*

Upon infection with *H. pylori*, cortactin not only undergoes CagA-mediated tyrosine dephosphorylation but also serine phosphorylation at S-405 and S-418 by PAK1 or ERK1/2 serine-threonine kinases. To this, phosphorylation at S-113 can be added, as was established using a set of highly specific phospho-antibodies that were specifically generated for this purpose [24]. Although the effect of *H. pylori*–dependent S-113 phosphorylation of cortactin by PAK remained unclear, it significantly reduced its binding to F-actin in cultured smooth muscle cells [24,35]. We observed a similar effect in infected AGS cells and originally speculated that *H. pylori* may trigger actin polymerization in infected AGS cells via N-WASP-dependent actin polymerization as well [24]. However, the opposite turned out to be the case. Surprisingly, we found that N-WASP and F-actin co-immunoprecipitated with cortactin in uninfected AGS cells, but this interaction was no longer observed in cells infected with *H. pylori* [24]. These results imply that infection by *H. pylori* triggers the dissociation of the cortactin/N-WASP complex, during which actin is released from the complex [24] (Figure 2C). Instead, cortactin phosphorylation by ERK at either S-405 or S-418, but not at both sites simultaneously, positively regulated the cortactin interaction with focal adhesion kinase (FAK) (Figure 2D). This interaction was clearly suppressed when cortactin was phosphorylated at Y-421, Y-466, and Y-482 [24]. It turned out that phosphorylation of S-405 is highly important, as it is essential for binding to FAK as well as for activation of its tyrosine kinase activity (Figure 2D). It is the interaction of cortactin with FAK that induces FAK phosphorylation at Y-397, Y-407, and Y-576, which highly increases its kinase activity in vitro and in vivo [24]. In addition, it was shown that integrin-mediated host cell adhesion to the extracellular matrix (including fibronectin binding) increases serine phosphorylation of cortactin, leading to subsequent FAK activation and conventional cell spreading [24]. It was also demonstrated, that this effect can be counteracted by transfected CagA-dependent activation of phosphatase SHP2, which downregulated FAK activity by its dephosphorylation [97].

Finally, as mentioned above, upon SDS-PAGE cortactin dissociates into two bands of 80 and 85 kDa (p80 and p85), as has been demonstrated for various cell lines, but it remained unclear what their phosphorylation states would be. Remarkably, infection of AGS cells by *H. pylori* was specifically associated with the serine phosphorylation of these two protein species. Upon infection, p80 was phosphorylated at S-113 and S-418, while p85 was phosphorylated at S-113 and S-405, as was shown for AGS cells [24]. This suggested a role for phosphorylation of S-405 in inducing a switch between the p80 and the p85 forms. However, various other modifications for such a switch have been put forward [23]. The relative abundance of these two cortactin forms seems to relate to tumor tissue. For instance, p80 dominates in various HNSCC cell lines, but upon treatment with epidermal growth factor (EGF) cortactin completely converts to the 85 kDa form within one hour [23]. Moreover, p85 cortactin is strongly expressed in colorectal cancer biopsies compared to neighboring tissue cells [101]. These observations led to the proposal that *H. pylori*-dependent phosphorylation of cortactin at S-405 and generation of p85 may induce a conformational change in cortactin and trigger downstream signal transduction events that are involved in the gastric carcinogenesis linked to *H. pylori* infection [24].

## 7. Role of Cortactin in VacA-Induced Apoptosis

The *H. pylori* toxin VacA has been shown to be involved in the disruption of mitochondrial dynamics, which subsequently led to apoptosis of gastric epithelial cells via activation of the pro-apoptotic Bcl-2-associated X (Bax) signaling factor [102,103]. In a more recent work, it was shown that cortactin may play a potential role in this scenario [98]. Acid-activated purified VacA induced apoptosis in wild-type AGS cells through a mitochondria-dependent pathway (Figure 2E). Increased expression of cortactin by lentiviral transduction of the gene led to significantly larger numbers of apoptotic AGS cells. Conversely, knockdown of cortactin using shRNA reduced the number of apoptotic cells. The expression levels of the pro- and anti-apoptotic proteins Bax and Bcl-2 were increased or decreased in transduced AGS cells versus wild-type control cells, respectively [98]. Moreover, in cortactin shRNA knockdown cells, the expression levels of Bax were downregulated, whereas Bcl-2 protein levels were upregulated. These data suggest that cortactin is somehow implicated in triggering cellular apoptosis induced by purified VacA. However, the exact mechanism and phosphorylation status of cortactin in the signal cascade remain unknown. It might be that cortactin plays a role during endosomal trafficking of VacA from the cell membrane to the mitochondria [104], but further investigations are clearly required to unravel the detailed function of cortactin in programmed cell death. In this regard, it is interesting to note that the bioactive compound quercetin from the plant *Polygonum capitatum* was shown to protect against gastric inflammation and apoptosis associated with *H. pylori* infection in mice by affecting the expression level of p38 MAP kinase, Bcl-2 and Bax [105]. Thus, quercetin can regulate the balance between gastric cell proliferation and apoptosis in the gastric epithelium and could be a novel candidate therapeutic agent to treat *H. pylori*-induced gastritis and other diseases.

## 8. Potential Role of Cortactin in Podosome and Invadopodia Formation

Previous studies have shown that infection by *H. pylori* may not only contribute to gastric diseases but may also contribute to various extra-gastric disorders including liver cirrhosis and hepatocellular carcinoma [106]. In this regard, it was demonstrated that infected primary hepatocytes isolated from mice were able to accumulate actin-rich cytoskeletal structures (called podosomes) at the ventral plasma membranes. These subassemblies encompassed proteolytic activity and were shown to be positive for specific podosome marker proteins including certain integrin members—vinculin and cortactin [106]. In general, it was described that podosomes may represent precursors of invadopodia, and the expansion of both structures could be important during epithelial-mesenchymal transition (EMT) and cancer cell progression in humans [107]. In recent studies, it has also been demonstrated that the actin-binding protein cofilin1 can induce EMT by promoting cytoskeletal rearrangements, while silencing of the cofilin1 gene leads to inhibition of EMT, invasion, and metastasis of gastric cancer cells [108]. Of special interest in this process is the Na+/H+ exchanger isoform 1 (NHE-1) protein, which plays an important role in regulating intracellular pH and osmotic homeostasis. In gastric cancer cells, NHE-1 expression is known to be upregulated and can affect cell migration and invasion [109]. Interestingly, cortactin–cofilin1 associations in the invadopodia are regulated by local pH changes mediated through NHE1. In fact, cortactin tyrosine phosphorylation at Y-421 and Y-466 enabled the recruitment of NHE1 to the invadopodia compartment, where it locally increased the pH to cause the release of cofilin1 from cortactin [110]. The released cofilin1 severs F-actin to generate free actin barbed ends and new filament elongation, which eventually induces invadopodial membrane protrusions [111]. Further studies should demonstrate the detailed role of *H. pylori* and its virulence factors in these processes.

## 9. Conclusions and Perspectives

Upon infection with *H. pylori*, a number of molecular pathways in a host cell undergo dramatic alterations, among which reorganization of actin cytoskeleton plays a crucial role to maintain chronic infection. By means of its T4SS, *H. pylori* injects proto-oncogenic CagA into gastric epithelial cells, which subsequently binds to and activates Csk, an inhibitor of Src. A molecular (Src-ERK-dependent) switch between tyrosine and serine phosphorylation of cortactin residues is the “Achilles heel” of a host cell, which *H. pylori* utilizes to its own benefit during infection [25]. The kinase FAK is being activated by S-405 phosphorylated cortactin, in particular the p85 form, which then influences focal adhesion turnover and the cell attachment that is of crucial importance in cell motility and elongation. When AGS cells are infected in vitro, they scatter and elongate simply because their lagging ends can no longer detach properly during migration due to enhanced FAK activity at these sites [24]. It is highly likely that the serine phosphorylation sites of cortactin function as the controller to regulate the activity of the SH3 domain that can mediate Arp2/3 complex–dependent actin polymerization. These steps form a signal transduction pathway that is able to explain the phenotypic outcome. The most important role of the tyrosine phosphorylation sites is that it suppresses this route.

Moreover, additional phosphorylation sites present in cortactin have recently been described that deserve attention. In particular, casein kinase (CK) 2α was shown to phosphorylate cortactin in HNSCC cells at the conserved threonine T-24 that resides at the Arp2/3 binding NTA domain [112]. This site may also be hijacked by *H. pylori*, as it has been shown that it can activate CK2α serine-threonine kinase to mediate T-24 phosphorylation in human gastric epithelial cells [113]. This opens the possibility that the dissociation of the cortactin/N-WASP/actin complex observed during *H. pylori* infection [24] can be explained by T-24 phosphorylation of cortactin by CK2α, although T-24 phosphorylation has not yet been demonstrated in gastric cells in vitro or in vivo. Other possible targets for *H. pylori* can be cyclin-dependent kinase 5 (CDK5) and protein kinase D1 (PKD1) that both can phosphorylate cortactin, respectively, at threonines T-145 and T-219 and at serine S-298 [30,37]. A potential role of these kinases in *H. pylori* pathogenesis or oncogenesis has not yet been investigated. Phosphorylation by the protein kinase C (PKC) at S-135, T-145, and S-172 located in the “cortactin repeats” was shown to mitigate both actin-binding and actin-crosslinking activity in SH-SY5Y neuroblastoma cells [114]. These three residues represent newly discovered phosphorylation sites, which are of interest to be investigated during *H. pylori* infection.

In conclusion, it is quite likely that cortactin-mediated regulation of cellular processes is even more complex than known to date, and additional phosphorylation sites can possibly be utilized by *H. pylori* that affects the cytoskeleton of host cells. Future studies will hopefully add new insights in a multifaceted cortactin regulation, which apparently has a number of yet unknown partners. The mechanisms through which *H. pylori* activates host cell movement and invasion would provide a deeper understanding of cancer development and metastasis originating from the gastrointestinal tract.

## Figures and Tables

**Table 1:** Reported interacting partners of cortactin and proposed functions in health and disease.

Phospho-Sites in Cortactin	Interaction Partners	Cortactin Kinase	Proposed Function	Host Cell	Applied Methods	References
unknown	WIP	unknown	Arp2/3 complex activation	HEK293	IP, WB, IFM, APA, GST-BA, YTHA	[27]
	Fgd1			In vitro	APA, GST-BA	[31]
				MC3T3-E1 (Mouse osteoblast cells) and COS-7 (monkey kidney fibroblast-like cells)	YTHA, GST-BA, IP, ICC, WB, IFM	[32]
	ZO-1		Cell-cell junction formation	*Drosophila melanogaster* (Canton-S wild type) embryo	YTHA, NB, GST-BA, WB, IFM, IP	[33]
	Shank		Synapse morphology and function	Dissociated hippocampal cultures	WB, ICC, CLSM, PALM, SMA	[34]
S-113	unknown	PAK1	Reduced binding of cortactin to F-actin	A7r5 (Pancreatic ductal adenocarcinoma cells)	In vitro KA, MS, GST-BA, IFM	[35]
S-298	WAVE2	PKD1	Generation of a 14-3-3 binding motif; binding to F-actin; Arp2/3 complex activation	Panc89 (PDAC), MCF-7 and HEK293T cells	IP, IHC, ABA, APA, CMA, GST-BA, In vitro ABA, CLSM, FRET, KA	[36]
	β-catenin and vinculin		Destabilization of adherence junctions	HEK293T and Caco-2 cells	IP, IHC, ABA, CLSM, IPA, FRET, CCAA,	[37]
S-405	FAK	ERK1/2	FAK activation; cell motility and elongation; p85 phenotype	AGS (human gastric adenocarcinoma cells)	IP, WB, In vitro KA, IFM	[24]
S-418	unknown		p80 phenotype			
	unknown		Co-localization with F-actin in invadopodia	UMSCC1 (Head and Neck Squamous Cell Carcinomas)	WB, IP, IHC, IFM, CMA	[23]
	unknown		Localization in lamellipodia	UMSCC2 (Head and Neck Squamous Cell Carcinomas)		
S-405, S-418	N-WASP		Lamellipodia dynamics and motility	HNSCC (Head and Neck Squamous Cell Carcinomas)		
	WAVE2	PKCδ	G-actin polymerization, F-actin stress fiber formation; cell migration	HASMC (Human Aortic Smooth Muscle Cells)	WB, IP, IFM, APA	[38]
Y-421	Gelsolin	Src	Increase of migratory capacity (migration and invasion; prometastatic, migratory phenotype	PDAC (Pancreatic ductal adenocarcinoma cells)	IHC, WB, PA, CMA, Scratch assay, CIA, MDA, IFM, IP	[39]
Y-421, Y-466	Adaptor protein NCK1		Nck1-dependent Arp2/3 activation	MDA-MB-231 (epithelial cell line from human breast cancer)	WB, IP, APA, FRET, LSM	[40]
	Vav2		Invadopodium maturation; actin polymerization, matrix degradation, and invasive migratory behavior	MDA-MB-231	WB, IP, GST-BA, IFM, APA, GST-BA, GEF AA, FRET	[41]
Y-421, Y-466, Y-482	unknown		Inhibition of cortactin’s F-actin cross-linking activity; Enhanced cell migration	ECV304 (Human endothelial cells)	CMA, AR, IP, CLSM	[42]
	Endothelial myosin light chain kinase (MLCK)		Inhibition of MLCK binding to F-actin; abolished cortactin-mediated augmentation of Arp2/3-stimulated actin polymerization	In vitro	GST-BA, WB, In vitro KA	[28]
	unknown	Abl	Dorsal-wave formation leading to lamellipodial protrusion	Mouse embryonic fibroblast	WB, IP, In vitro KA	[43]
Y-499	Arp2/3	Srk	Filopodia formation	Bag cell neurons from *Aplysia californica*	ICC, IFM, STORM, WB, In vitro KA	[44]
T-145, T-219	Dynamin	CDK5	Inhibition of actin-bundling activity; reduction of pseudopodal formation	NG108-15 (glioma-derived cells)	CMA, In vitro KA, ABA, GST-BA, WB, EM, MS	[30]

## Abbreviations

ABA	F-actin binding assay
APA	Actin polymerization assay
AR	Autoradiography
CCAA	Cell–Cell Adhesion Assays
CIA	Collagen invasion assay
CLSM	Confocal Laser Scanning Microscope
CMA	Cell motility assay
EM	Electron microscopy
FRET	Fluorescence Resonance Energy Transfer assay
GEF AA	GEF activity assay
GST-BA	GST-binding assay
ICC	Immunocytochemistry
IFM	Immunofluorescence Microscopy
IHC	Immunohistochemistry
In vitro	In vitro actin branching assay
In vitro KA	In vitro Kinase Assay
IP	Immunoprecipitation
IPA	Intestinal permeability assay
KA	Kymograph Analysis
LSM	Laser Scanning Microscope
MDA	Matrix degradation assay
MS	Mass Spectroscopy
NB	Northern Blot
PA	Proliferation assay
PALM	Single-Molecule Photoactivated Localization Microscopy
STORM	Stochastic Optical Reconstruction Microscopy
SMA	Synapse Morphology Analysis
WB	Western Blotting
YTHA	Yeast Two-Hybrid Analyses
